# Single-rater reliability of a three-dimensional instrument for decision-making in tertiary triage and ICU- prioritization—a case vignette simulation study

**DOI:** 10.1186/s12871-023-02173-2

**Published:** 2023-06-20

**Authors:** Stefan Bushuven, Michael Bentele, Bianka Gerber, Andrej Michalsen, Ilhan Ilkilic, Julia Inthorn

**Affiliations:** 1 Institute for Hospital Hygiene and Infection Prevention, Hegau-Jugendwerk Hospital Gailingen, Health Care Association District of Constance, Hausherrenstrasse 12, 78315 Radolfzell, Germany; 2 Institute for Medical Education, University Hospital, LMU Munich, Munich, Germany; 3 Institute for Anesthesiology, Intensive Care, Emergency Medicine and Pain Therapy, Hegau Bodensee Hospital, Singen, Germany; 4 Department of Anesthesiology, Critical Care, Emergency Medicine and Pain Therapy, Konstanz Hospital, Constance, Germany; 5 Istanbul University, Istanbul, Turkey; 6 Center for Health Care Ethics, Hanover, Germany

**Keywords:** Pandemic, ICU, Tertiary triage, Ethics, Prioritization, Algorithm

## Abstract

Disconcerting reports from different EU countries during the first wave of the COVID-19 pandemic demonstrated the demand for supporting decision instruments and recommendations in case tertiary triage is needed. COVID-19 patients mainly present sequentially, not parallelly, and therefore ex-post triage scenarios were expected to be more likely than ex-ante ones. Decision-makers in these scenarios may be highly susceptible to second victim and moral injury effects, so that reliable and ethically justifiable algorithms would have been needed in case of overwhelming critical cases.

To gather basic information about a potential tertiary triage instrument, we designed a three-dimensional instrument developed by an expert group using the Delphi technique. The instrument focused on three parameters: 1) estimated chance of survival, 2) estimated prognosis of regaining autonomy after treatment, and 3) estimated length of stay in the ICU. To validate and test the instrument, we conducted an anonymous online survey in 5 German hospitals addressing physicians that would have been in charge of decision-making in the case of a mass infection incident. Of about 80 physicians addressed, 47 responded. They were presented with 16 fictional ICU case vignettes (including 3 doublets) which they had to score using the three parameters of the instrument.

We detected a good construct validity (Cronbach’s Alpha 0.735) and intra-reliability (*p* < 0.001, Cohens Kappa 0.497 to 0.574), but a low inter-reliability (*p* < 0.001, Cohen’s Kappa 0.252 to 0.327) for the three parameters. The best inter-reliability was detected for the estimated length of stay in the ICU. Further analysis revealed concerns in assessing the prognosis of the potentially remaining autonomy, especially in patients with only physical impairment.

In accordance with German recommendations, we concluded that single-rater triage (which might happen in stressful and highly resource-limited situations) should be avoided to ensure patient and health care provider safety. Future work should concentrate on reliable and valid group decision instruments and algorithms and question whether the chance of survival as a single triage parameter should be complemented with other parameters, such as the estimated length of stay in the ICU.

**Supplementary Information:**

The online version contains supplementary material available at 10.1186/s12871-023-02173-2.

## Introduction

### Background

Since 2019, SARS-CoV-2 [1] has caused a pandemic challenging health care systems worldwide. As of the 2^nd^ of April 2023, more than 760 million people were infected, and an estimated 6.8 million have died of COVID-19 worldwide. Many more non-COVID victims were affected due to the overwhelmed medical infrastructure with delays in medical treatment, especially in the first wave which resulted in changes in emergency management [2] with patient relocation between regions and nations (e.g., the “Cloverleaf System” in Germany) [3]. Scarcity of resources, i.e., personnel, material, and time played a significant role in many countries leading to the challenge of ensuring critical care capacity for all patients in need. The requirement of “tertiary triage” [4, 5] and the development of recommendations for prioritization by national societies, and therefore the demand for preparing triage protocols and training, was pressing in most European hospitals. This, too, was the case in Germany and Switzerland, which lead to the development of recommendations by DIVI [6] and SAMW [7].

In a prioritization process, distributors of medical resources rely on rational decision-making, expert medical knowledge, assessment and comparison of prognoses, and transparent ethical criteria with a theoretical foundation [8]. In battlefield and emergency medicine, with the contemporaneous occurrence of many victims, primary triage is needed. In contrast, during the COVID-19 pandemic, patients with acute respiratory distress syndrome (ARDS) mainly developed and still develop these symptoms sequentially with the need for secondary triage in emergency departments (EDs) and tertiary triage in intensive care units (ICUs) [9]. Thus, there might be more time for ICU specialists to decide whom to treat in an ICU. In Germany, the discussion is aggravated by issues resulting from the ex-ante (two persons compete for the last free bed) and ex-post (a person with a better prognosis arrives later and competes for a bed occupied by a patient with a worse prognosis than the new patient) situations leading to a substantial (and unresolved) discourse in German politics, jurisdiction, ethics, and medicine [10–15]. The German recommendations to aid decision-making in resource allocations in the COVID pandemic were recently challenged in court. Subsequently, the German supreme court ruled the German parliament to enact a law to prevent discrimination against patients with disabilities in prioritization scenarios [BGH 1 BvR 1541/20, December 16, 2021]. This resulted in a legal regulation as part of the infection protection act requiring further discussion [16].

The fair distribution of ICU resources, such as treatment spaces and ventilators, were a commonly described problem during the pandemic. Thus, there was rising interest in how to distribute these resources. Most instruments, including the German one [6], followed a utilitarian approach to save as many lives as possible. Furthermore, using short-term survival as the main criterion was questioned in several ways, as survival alone and as a dichotomous issue does not always mean surviving in a desirable qualitative status respecting the bioethical principles (respect for autonomy, beneficence, non-maleficence, justice) [17].

Next, the estimated length of stay in the ICU could also play a role, as patients with COVID-19 may require intensive care for many weeks. In contrast, patients admitted after major surgery (e.g., aortic aneurysm repair), an uncomplicated myocardial infarction, or stroke may only need some days or even hours in the ICU. Consequently, the estimated time spent in the ICU could be seen as an allocation factor and would lead to the same objective to save as many lives as possible. Accordingly, the chance of survival must be questioned as the single dichotomous parameter, as surviving in the desired state and time spent in the ICU may be further parameters influencing the decision-making process.

### Rationale

The aim of this study was to investigate the feasibility, validity, and intra- and inter-reliability of a newly developed three-dimensional instrument for tertiary triage decisions. This instrument applies especially—but not exclusively – to ex-post scenarios. The three dimensions were (1) chance of survival, (2) chance for autonomous decision-making after therapy in the ICU, and (3) length of stay (LOS) in the ICU.

We hypothesized that a three-dimensional instrument developed by a team of medical experts and tested in a simulation of a mass casualty event in the pandemic is valid (H1) as well as intra- (H2) and inter-reliable (H3).

## Methods

### Study design and setting

We conducted the study in following steps:

1. Iterative Delphi procedure (5 ICU physicians)
2. Development of case vignettes (same group)
3. Development of the questionnaire (same group)
4. Pretesting of the questionnaire
5. Revision of the questionnaire
6. Distribution of the anonymous questionnaire (approximately 80 physicians)

The hypothetical instrument with the three main dimensions was developed parallel to the publication of triage recommendations by German medical societies and guidelines by scientific associations [6], which would have been used in a real scenario.

The instrument was developed using an iterative modified online-based Delphi procedure with members of the ethical committees of the five hospitals. Additionally, the group developed a phase-based model to justify the potential implementation of the instrument. According to this model, the instrument would be used only in phase 3 of a pandemic (absolute shortage of resources), but not in phase 1 (regular work) or phase 2 (relative shortage of resources).

The Delphi group consisted of five critical care physicians with subspecialties in infectious diseases, neurology, pulmonology, emergency medicine, and palliative care. Taking an iterative approach, these persons created an instrument using the three dimensions: chance of survival, respect for autonomy, and estimated length of stay in the ICU. From March to May 2020, we developed and conducted an explorative cross-sectional anonymous online survey among intensive care physicians in five German hospitals. In the first step, we designed thirteen fictitious clinical case vignettes of critical care patients and tested for content validity in a validation group. Three cases were doubletted for intra-reliability testing—providing 16 vignettes altogether. Second, the survey was pretested for face-, content-, and construct-validity and reliability in this validation group of four clinicians. Third, the fully developed questionnaire was provided to participants. Validation group members were excluded from the final questionnaire.

The survey was provided by Enuvo GmBH Zuerich, Switzerland. IP addresses were blinded towards the investigators. Therefore, it was not possible to retrace survey answers to participants.

The data was analyzed using Microsoft Excel, XL-Stats (Fa. Addinsoft) and SPSS 25.0 (IBM).

The study results were used in the Master of Medical Ethics program of the Gutenberg University of Mainz with the permission of the medical directors of participating hospitals and was supervised by two educators and certified specialists for medical ethics. According to the relative legal stipulations, no ethical approval was needed for this anonymous survey.

### Participants

Participants were consultants or trainees in neurology, critical care, anesthesiology, emergency medicine, palliative medicine, or internal medicine. The survey was sent to 80 physicians in five hospitals in southern Germany. All participants held positions in their hospitals eligible for involvement in decision-making in a triage situation.

### Variables

Aside from demographic data (age, profession, working experience), each case vignette was assigned three parameters addressing 1) estimates of survival chance, 2) chance of rehabilitation, and 3) LOS in the ICU. Participants chose between the four options in each parameter, as shown in Table 1.

**Table 1:** Developed Instrument

	Estimated survival with access to critical care “SURVIVAL”	Estimated best prognosis after rehabilitation concerning autonomy and free will “AUTONOMY”	Estimated length of stay in ICU “LOS”
0	Very likely e.g.: intermediate care after surgery, paraplegia, intoxication, AV-Blockade before pacemaker implantation, uncomplicated myocardial infarction, mild stroke	Complete Autonomy Complete mental recovery is anticipated	Up to 48 h
5	Likely Subdural haemorrhage, traumatic brain injury, pneumonia, Post-ROSC with awakening after CPR, repair of aortic aneurysm, gastrointestinal bleeding, hyperkalaemia in renal impairment	Incomplete Autonomy Predominant mental recovery is anticipated with temporary legal supervision	3- 9 days
20	Intermediate Sepsis, ARDS, AECOPD, severe trauma, severe Stroke, oncologic diseases with an overall prognosis to survive more than 1 year, liver cirrhosis Child A/B, heart failure, shock, severe traumatic brain injury	Incomplete Heteronomia High risk for insufficient mental recovery with long term legal supervision	10–25 days
30	unlikely Post-ROSC with cerebral edema, severe subarachnoid bleeding (Hunt&Hess V), severe oxygen dependent COPD, Frailty, oncological diseases with anticipated survival of less than 2 years, liver cirrhosis Child C, hypoxic encephalopathy	Complete Heteronomia The patient will be under legal supervision for the rest of his life and will not be able to create or express his free will	25 days or more

### Expected biases

We addressed the selection bias by choosing a pre-selected group of physicians likely to be involved in possible tertiary triage situations during resource scarcity. Thus, we addressed both specialists and trainees of different ICU specialties As case vignettes are a surrogate of patients, there might be a recall bias to prior experiences of the participants. To lower this bias, we presented the physical information using the “ABCDE” and “SAMPLE” mnemonics used in emergency medicine and critical care [18]. Another bias concerning the response rate might be the COVID-pandemic itself with high awareness of the situation and thus possible a better response rate. The sample size was small [19], but representability was acceptable as the study population covered ICU physicians in the addressed with a response rate of more than 50 percent. Generalizability for national or international level was not part of our study. As decision-making in this conflicting process might trigger absenteeism, questions were designed to be mandatory and could not be skipped.

### Study size

Participants were informed about the study three times via mailing lists to get a response from more than 50 percent of all physicians potentially involved in triage situations in the participating hospitals.

### Quantitative variables

A translation of the German case vignettes are shown in Additional file 1. An overview of cases is given in Table 2 with one translated detailed case in Table 3. For each case, participants were asked to assign the four grades according to each parameter (survival, respect for autonomy, LOS) with 0, 5, 20 and 30 points, with best prognosis being zero points in each parameter. The point allocation was approved by the Delphi expert group.

**Table 2:** Patient vignettes (short version)

**Case Vignette**	
**1**	**67, male retired bus driver, ARDS, COVID-19, cardiogenic shock, smoker, carotid stenosis, patient decree unavailable, full therapy. Patient is mechanically ventilated in ICU with low-dose catecholamine therapy**
2	**55, female vendor, fronto-basal meningioma, compression of fourth ventricle, epilepsy, pre-surgery but cerebral herniation imminent, ICU needed post-operatively, not yet in ICU**
3	**45, male teacher, ARDS, COVID-19, infected by student, stable in the ICU, extubation planned**
4	**88, female “crazy cat lady”, femoral neck fracture, chronic heart failure, dementia, post operation, no PACU available**
5	**34, male mechanic, suicide attempt by jumping off a roof top (10 m), pneumothorax, severe traumatic brain injury with cerebral edema and signs of mesencephal inherniation, splenic rupture, open femur fracture, fracture of dens axis, fracture of fifth lumbal vertebrae with rupture of the spinal cord, hemorrhagic shock, ARDS. Mechanically ventilated with high-dose catecholamine therapy. Patient scheduled for emergency operations**
6	**66, female, sudden cardiac arrest by STEMI, lay rescuer CPR (45 min), cardiogenic shock, hypothermia, transported by EMS to the ICU, cath-lab planned in 10 min, mechanical ventilation**
7 ( 1)	**67, male retired bricklayer, ARDS, COVID-19, cardiogenic shock, smoker, carotid stenosis, patient decree unavailable, full therapy. Patient is mechanically ventilated in ICU with low-dose catecholamine therapy [Case Doublette, #1]**
8	**68, male, retired person, intracerebral haemorrhage into the basal ganglia, ventilated for 12 days, otherwise stable, neurological rehabilitation planned, family members insist on full therapy**
9	**28, female, ARDS, COVID 19, infected by toddler via kindergarten,** **morbid obesity, ventilated for 14 days, 100% oxygen, ECMO not available**
10 ( 3)	**44, male teacher, COVID-19 ARDS, intubated 4 days ago. [Case Doublette, #3]**
11	**51, female police officer, severe peritonitis after colon resection due to colon cancer, septic shock with acute renal injury and daily dialysis, abdominal wound dehiscence, mechanical ventilation**
12 ( 8)	**68, male pensioner, intracerebral haemorrhage into the basal ganglia, ventilated for 12 days, otherwise stable, neurological rehabilitation planned, family members insist on full therapy [Case Doublette, #8]**
13	**61, male industrial clerk, aortic aneurysm (6.8 cm), severe peripheral occlusive artery disease, hypoventilation syndrome, chronic pulmonary obstructive disease, diabetes type 2, operation planned tomorrow**
14	**31, female, severe trauma 5 years ago, minimal conscious state, dysfunction of ventriculo-peritoneal shunt system, epilepsy, tetraspastic with baclofen pump, tracheostoma, revision of VP shunt planned with need for 1 day ICU care postoperatively**
15	**61, male pharmacist, severe progressive amyotrophic lateral sclerosis. Bacterial pneumonia with acute respiratory failure, CPAP home therapy, progressive respiratory failure, wants to survive with tracheostomy to finish writings with computer based ocular assistance**
16	**38, male, Down syndrome, COVID 19 pneumonia, severe respiratory failure (nasopharyngeal tube in place), intubation and mechanical ventilation needed in ICU**

**Table 3:** Detailed case vignette for Case 1 (translated from German)

**CASE 1** You are caring for the 67-year-old retired bus driver Johann Perler (110 kg, 187 cm) suffering from a COVID-19 infection
**Airway:** intubated 8 days ago due to ARDS, difficult intubation **Breathing:** FiO2 90%, Pinsp 30 cmH2O, PEEP 15 cmH2O, intermittent prone positioning **Circulation:** Shock (low-grade therapy with Norepinephrine), intermittent atrial fibrillation and irregular supraventricular tachycardia requiring cardioversion once **Disability**: none, RASS -5 due to sedation **Exposure:** central line, arterial cannula, endotracheal tube
**Signs & Symptoms:** Onset of symptoms 14 days ago with high fever, infection via his spouse, admitted 8 days ago to ICU with respiratory distress due to ARDS **Allergies:** none **Medication:** Chronic prescription: Valsartan and acetylic acid. Medication in ICU: propofol, sufentanil, piperacillin/tazobactam, pantoprazol, enoxaparine low dose **Past Medical History:** thromboembolic stroke 5 years ago, carotis disobliteration 5 years ago, smoker (50 pack years) **Last Meal:** enteral feeding via nasogastric tube **Events:** none
**Chest CT:** Opaque infiltrations in both lungs, basal atelectasis, unknown mass in the right upper lobe **Echocardiography**: sufficient left-ventricular function, dyskinesia of the anterior wall
**ABG**: pH 7.4, pO2 67 mmHg, pCO2 45 mmHg, SaO2 95%, Na 140 mmol/l K 4,5 mmol/l, lactate 1,7 mmol/l **Blood Sample Test**: WBC 6,7 cells/mm^3^, Hb 13,4 g/dl, Platelets 515,000/mm^3^ **Serum**: Creatinine 2.4 mg/dl, AST 67 U/l ALT 77 U/L, GGT 123 U/l, Bilirubin 1.2 mg/dl, C-reactive protein 24 mg/dl, Procalcitonin 3.4 pg/ml
**Patient will**: unknown **Family**: Full Therapy **Plan**: stabilize oxygenation, obtain informed consent for tracheostomy, nephrology consultation

### Statistical methods

Data analysis comprised extensive measurements of construct validity (Bartlett Sphericity, Kayser-Mayer-Olkin coefficient, Cronbach’s Alpha, Gutmann criteria and Split-Half Reliability). Bartlett sphericity below 0.05 or KMO above 0.5 were suggested to be substantial collinearity, excluding further tests. Cronbach’s Alpha and Gutman criteria (Lambda 2,3,4,5) above 0.6 were considered to show sufficient construct validity of the survey. Subgroup analyses were conducted using non-parametric tests due to the small sample size and absence of normal distribution. Intra-Reliability (ratings of the same participants) of doublet cases were measured by Cohen’s Kappa, inter-reliability (different participants’ rating of the same case) for all cases with Fleiss’ Kappa. The interpretation was accomplished according to Landis (0.0 -0.2 no, 0.2–0.4 mild, 0.4–0.6 moderate, 0.6–0.8 strong, and above 0.8 perfect agreement of the participants or between ratings on a similar case by one participant).

### Qualitative variables

Free-text entries at the end of the survey were analyzed according to Bradley, taking a single coder approach [20]. The coder is the corresponding author of this work with prior experience in qualitative research.

## Results

### Participants and descriptive data

Of the 80 physicians addressed, 47 responded to the questionnaire (58.8%), with 29 of this group completing the survey (61.7% of the responders and 36.25% of all persons addressed). Of all 47 participants, 36 (76.6%) were physicians with completed specialty education. Most of the participants (36) were anesthesiologists and critical care physicians (76.6%), five were internal medicine physicians (10.6%), four surgeons (8.5%), and 2 (4.2%) were other specialized physicians (e.g., neurology). 35 had completed additional qualifications in emergency medicine (74.5%), 17 in critical care medicine (36.2%), and 6 in palliative care (12.8%).

### Main results

Hypothesis 1: Reliability coefficients according to Gutmann showed Lambda-values of 0.719 (λ1), 0.793 (λ2), 0.735 (λ3, Cronbach’s Alpha), 0.575 (λ4, Split-Half), and 0.757 (λ5). Lamba-2 was barely missed but is sufficient for group evaluations [21]. Cronbach’s Alpha was sufficient. Split half was also barely missed but is explained by the small sample size. The primary factor analysis with Varimax rotation showed Eigenvalues of more than 1, with 15 factors explaining 88.3% of the complete variance.

Hypothesis 2: Intra-reliability for doubletted cases 1/7, 3/10, and 8/12 showed a Cohen’s Kappa of 0.574, 0.497, and 0.505 for survival; 0.652, 0.63, and 0.69 for autonomy; and 0.536, 0.28, and 0.742 for LOS. Each test parameter was highly significant (*p* < 0.001).

Hypothesis 3: Inter-reliability was highly significant for all three parameters (*p* < 0.001), comprising a satisfying kappa of 0.252 for survival, 0.312 for autonomy, and 0.327 for LOS in ICU.

Operationalization of the score, with the assignment and summation of points (0–30), was highly significant but not reliable (Kappa = 0.126).

One case (Case 15: progressive amyotrophic lateral sclerosis with pneumonia) raised concerns about the validity of the autonomy parameter. Although autonomous decision-making would be possible in this patient, some participants assigned a poor prognosis for autonomy. The rating of poor prognosis also occurred in cases 4, 14, and 16 with preexisting disease.

### Subgroup analysis

The Kruskal Wallis-tests for subgroup analysis revealed no significant difference between cases or respondent subgroups with the following exceptions:

In case 9 (young women with COVID-19), specialists estimated a longer ICU LOS than physicians in training (*p* = 0.014). There was no difference concerning physicians’ answers with or without an additional emergency medicine qualification.

However, there were differences noted between specialists with an additional critical care qualification and those without. For example, specialists estimated a longer ICU LOS for case 12 (basal ganglia bleeding, but not in the doublet case 8), while in case 5 (suicide attempt) ICU specialists assigned a worse prognosis concerning survival (*p* < 0.05 each).

### Qualitative codings

The analysis of free-text entries at the end of the questionnaires revealed two main themes:

First, most participants stated that substantial uncertainty remained for estimations of prognoses with a need for multi-rater approaches (“*Difficult. We should always decide in teams*”, “*We cannot estimate the fate of single persons. It stays individual*”, “*Difficult to get moral and factual in coexistence*”, “*It depends on own experiences*”). Second, prioritization instruments or algorithms are necessary only for extreme situations. However, in these situations without alternatives, it would be accepted (“*We need such scores when in doubt.”* “*We have to be desperate to need this*”, “*It’s difficult. But in these situations, there would be no alternative*”).

## Discussion

### Key results

In this multicenter study using fictional case vignettes, we were able to show that the instrument was valid (confirming H1) and intra-reliable (confirming H2), but not sufficiently inter-reliable (rejecting H3), especially when summative scoring points were used.

To our knowledge, this is the first study testing for inter-reliability in tertiary triage. It provides essential information for ongoing research and medical education regarding this topic. Further, our results indicate the imperative demand for multi-person decision-making in tertiary triage due to limited inter-reliability in the prognostic estimation of ICU patients—as tested with the patient case vignettes.

### Biases

Selection bias is a limiting factor for all questionnaires because only motivated persons participate and complete these surveys. In this study, we reached more than 50% of our target group potentially involved in triage processes, and more than a third of all addressed persons completed all of our questions. As one cannot differentiate the “non-responders” that were not reached by recruiters from those that refuse to participate, we could not estimate how many “non-responders” fall into these two subgroups. This is also the case for responders that did not complete the survey (“Drop-outs"), as we do not know why they quit the survey (e.g., because of response burden, technical issues or because of emotional factors by triage leading to “survey absenteeism”). Consequently, future research may evaluate why people might refuse to participate in ethical surveys on this topic like this. However, the reasons for not-responding or dropping out at this point of or research are unknown and therefore speculative.

With respect to a proportion of non-responders and “drop-outs” as a possible origin of error, and as our study was intended for hypothesis generation rather than epidemiological description, the representability of the sample, or at least the transferability of the results, can be considered acceptable for first tests on a new instrument. However, further research in other and larger target populations [22] is necessary, especially considering that generalizability could not be evaluated with this study concept. Furthermore, the selection bias might even be lower because social desirability and the uprising first wave of the COVID-19 pandemic may have stressed the possible demand for a triage instrument, increasing the motivation to participate but maybe leading to more drop-outs.

Second, our survey showed an overproportioned participation of anesthesiologists. Compared to other countries, Germany lacks its own critical care specialty, and anesthesiologists commonly also work in ICUs, not only in the operating theater. Concerning the comparable proportion of anesthesiologists in the five hospitals’ ICU rosters then, “local” generalizability showed to be reasonable and valid for real-life scenarios.

Recall-bias occurs if similar cases with different outcomes were experienced prior to the survey and may lead to different decisions. However, this could happen in reality as well and should be assessed in future projects. Gender and in-group biases play an ambiguous role in triage in emergency departments [23, 24]. In this study we did not obtain those main demographic parameters to guarantee anonymity within the small sample size. We cannot assess these biases further here.

The response burden may have played a role as the survey is rather long with 16 complex cases each requiring decision-making. Normally, surveys should take a maximum of 20 min to complete. In this survey, participants took 7 min to 6 h (mean 48 min, standard deviation 68 min). Drop-outs stopped the survey after 6 min (mean value, min 0 min, max 48 min). Hence, the response burden may have played a role, too.

### Reliability

Inter-Reliability in this survey was moderate and lower compared to other studies in neuro-pediatrics [19], neurology [25], palliative care [26], and geriatrics [27]. However, despite manifold publication on prioritization in ICUs, we did not find any studies concerning the reliability of tertiary triage in a PubMed and Google Scholar search, although there is some evidence for primary and secondary triage using other emergency algorithms [28, 29].

This low reliability of single raters and the unavailability of algorithms for tertiary triage shows the demand for multi-rater discussions of prognoses, interprofessional shared decision-making, and when in doubt using structured ethical case conferences. National recommendations explicitly call for these case discussions with at least three persons (two experienced physicians, one nurse, and optionally one specialist in medical ethics) [6].

Whereas the parameters “survival” and LOS showed adequate reliability and validity, the parameter “autonomy” raised concerns for validity as some participants assigned a poor prognosis that would be expected for physical, but not psychological prognosis (amyotrophic lateral sclerosis case). Additionally, persons with preexisting limitations of autonomy (geriatric case, prior traumatic brain injury, trisomy 21) would be categorically disadvantaged if our instrument would be used. As a further limitation of this parameter, the patient’s will about his or her autonomy might differ substantially from the suggested autonomy by physicians. Therefore, anticipation of individual autonomy by other persons not knowing the victim and with no or limited access to information about the life and values of the persons (e.g. provided by family members) may be too “transcendent” to be reliably operationalized. Moreover, in triage situations the parameter and the will of the person might be reduced to a pure reconstructive state and consequently loosing its validity and impact in differentiation.

Thus, for these cases, the score showed adequate reliability but would not be valid and neither ethically nor legally justifiable for these groups due to the potential discrimination [30].

### Interpretation

Single-rater tertiary triage is susceptible to invalid decision-making and thus should be conducted by interprofessional shared decision-making [31] with several experts from different disciplines if possible. Triage decisions made by single raters may be erroneous or can be interpreted differently by other physicians leading to the possibility of moral distress and moral injury [32], second victim effects [33], and even forensic consequences.

In contrast to primary triage (with additional time pressure, e.g., in mass casualty incidents), interprofessional group decisions are realizable in tertiary triage when more time is available. Whereas “chance of survival” and “length of stay in the ICU” showed to be parameters with good validity and reliability, the parameter “respect for autonomy” did not. This was especially the case for patients with preexisting comorbidities and physical impairment who maintained psychological abilities. However, length of stay combined with the SOFA-score showed prognostic properties in research of other groups [34], so this parameter should be focused on in future research.

Physicians are trained to take measures to improve the health status of patients and to thereby reach an appropriate quality of life as to their own assessment. The possibility to return to a self-determined life should play a central role in therapy decisions and patient discussions. The parameter "autonomy" is intended to take this into account. The results show that this consideration, which is established in other everyday clinical practices, must be viewed critically when in need of triage, especially the aspects of non-discrimination and differing assessments by professionals. Respect for autonomy and individual assessment of future quality of life is of high ethical importance and should be an integral part of decision-making, including other means, e.g., living will or advanced care planning.

In this study, we did not include common scoring tools like SAPS or SOFA. These scores were developed for quality measurements and study protocols purposes or for detecting patients at risk for certain illnesses (e.g., sepsis), but not for tertiary triage in a complex setting of patients with different pathologies [35]. Due to the criticism of these scores for triage (leading to over- or under-triage) [36, 37], they should be used with caution – especially when comparing patients with different illnesses and comorbidities. Nevertheless, further studies can be helpful to show how these scoring – systems (and potentially the support by augmented intelligence and machine learning [38]) may impact human decision-making in single raters or ICU teams.

Our results indicate the need for further national and international research on this topic, concentrating on validity and reliability. Although the case vignettes show good transferability into other settings (e.g., communication training) their validity and reliability in triage instruments should be compared to real-life situations and patients.

Additionally, some respondents’ answers concerning autonomy show possible signs of a disambiguation of “autonomy” as “non-disability”. Consequently, medical professionals need to improve their knowledge competencies in medical ethics and clinical decision-making to prevent or ameliorate ableism or discrimination. In other words: even if a rational interprofessional decision-making process in a team is possible, it may be biased by false concepts when autonomy is regarded as a relevant parameter. Furthermore, it might be possible that responders’ differences in knowledge of the term “autonomy” and its interpretation are responsible for the poor inter-reliability of this parameter in our study. Consequently, further studies on triage instruments and algorithms like in this study should include knowledge assessments for this parameter and detect systematic bias in decision-makers.

## Conclusion

The three-dimensional instrument for tertiary triage tested among 47 physicians of different specialties did not meet the expected quality criteria for inter-reliability. Subsequently, single rater tertiary triage should be avoided whenever possible to maintain patient safety and forensic and psychological safety for decision-makers. For medical education and clinical decision-making, our findings indicate the need for training in prognostication and interprofessional shared decision-making.

## Supplementary Information

**Additional file 1.**

## Data Availability

The data sets generated and analysed during the current study are not publicly available due to internal policies and comprising mainly German items with need for intensive translation, re-translation and validation processes in the qualitative sections. Relevant Data is available from the corresponding author on request.
