# Supplementary material for: Single-rater reliability of a three-dimensional instrument for decision-making in tertiary triage and ICU- prioritization—a case vignette simulation study
Source: BMC Anesthesiol. 2023 Jun 20;23:215. doi: 10.1186/s12871-023-02173-2 (PMC10280907; doi:10.1186/s12871-023-02173-2)
Supplement: Supplementary file 1 — Additional file 1. [file 12871_2023_2173_MOESM1_ESM.docx]

**Appendix 1**

**CASE 1**

You are caring for the 67-year-old retired bus driver Johann Perler (110 kg, 187 cm) suffering from a COVID-19 infection.

Airway: intubated 8 days ago due to ARDS, difficult intubation
Breathing: FiO2 90%, Pinsp 30 cmH2O, PEEP 15 cmH2O, intermittent prone positioning
Circulation: Shock (low-grade therapy with Norepinephrine), intermittent atrial fibrillation and irregular supraventricular tachycardia requiring cardioversion once
Disability: none, RASS -5 due to sedation
Exposure: central line, arterial cannula, endotracheal tube

Signs & Symptoms:

Onset of symptoms 14 days ago with high fever, infection via his spouse, admitted 8 days ago to ICU with respiratory distress due to ARDS

Allergies: none
Medication: Chronic prescription: Valsartan and acetylic acid. Medication in ICU: propofol, sufentanil, piperacillin/tazobactam, pantoprazol, enoxaparine low dose
Past Medical History: thromboembolic stroke 5 years ago, carotis disobliteration 5 years ago, smoker (50 pack years)
Last Meal: enteral feeding via nasogastric tube
Events: none

Chest CT: Opaque infiltrations in both lungs, basal atelectasis, unknown mass in the right upper lobe

Echocardiography: sufficient left-ventricular function, dyskinesia of the anterior wall
ABG: pH 7.4, pO2 67 mmHg, pCO2 45 mmHg, SaO2 95%, Na 140 mmol/l K 4,5 mmol/l, lactate 1,7 mmol/l
Blood Sample Test: WBC 6,7 cells/mm³, Hb 13,4 g/dl, Platelets 515000/mm³
Serum: Creatinine 2.4mg/dl, AST 67 U/l ALT 77 U/L, GGT 123 U/l, Bilirubin 1.2 mg/dl, C-reactive protein 24 mg/dl, Procalcitonin 3.4 pg/ml

Patients will: unknown
Family: Full Therapy
Plan: stabilize oxygenation, obtain informed consent for tracheostomy, nephrology consultation

**CASE 2**

You are caring for a 55-year-old saleswoman Luisa Kerns (55 kg, 168 cm) with a large frontobasal meningioma and incipient compression of the 4th ventricle.

Airway: free

Breathing: free on room air

Circulation: stable

Disability: homohyme hemianopsia, for 3 weeks epileptic seizures due to brain tumour with perifocal edema, vomiting since several hours, ventricular drainage is done for acute relief, brain tumor surgery planned

Exposure: no special features

Signs & Symptoms: for 3 weeks epileptic seizures and increasing hemianopsia

Allergies: Ampicillin

Medication: Levetiracetam (2x1000 mg, for 3 weeks), Dexamethasone (4x4 mg)

Past Medical History: caesarean section, otherwise no previous diseases

Last Meal: 2 hours ago

Events: none

Chest CT: not performed

Echocardiography: not performed

ABG: not performed

Blood count: WBC 7,3 cells/mm³, Hb 12,1 g/dl, platelets 317000/mm³

Serum: creatinine 0.6mg/dl, GOT 44 U/l GPT 54 U/L, GGT 56 U/l, bilirubin 0.6 mg/dl, CRP 0.5 mg/dl, PCT 0.3 pg/ml.

Patient directive: Do no resuscitate

Relatives: none

Plan: tumor surgery with evacuation and revision of the fronto-base, High blood loss intra-operatively expected, expected 3 days intensive care stay according to NCH, postponement of surgery not possible due to impending loss of vision and onset of intracranial pressure symptoms.

**CASE 3**

You provide care to 45-year-old teacher Lukas Hammer (68 kg, 182 cm) with COVID 19 infection.

Airway: Intubated 4 days ago for ARDS.

Breathing: FiO2 40%, Pmax 22, peep 7, intermittent prone positioning

Circulation: stable

Disability: no restrictions, currently RASS -3 due to sedation, weaning process started

Exposure: central venous line, arterial line, intubation

Signs & Symptoms: onset 4 days ago with fever and cough, infection via colleagues in emergency care, referral via ED for ARDS

Allergies: none

Medication: no home medication except salbutamol spray if needed, propofol (current), piritramide (current), pantoprazole (current), enoxaparin low dose (current)

Past Medical History: inguinal hernia, allergic asthma (especially in spring months).

Last Meal: enteral nutrition

Events: none

CT chest: Opaque infiltrations in both lungs

Echocardiography: good LV function

ABG: pH 7.36 pO2 88 mmHg, pCO2 41 mmHg, SaO2 99%, Na 139 mmol/l K 4.2 mmol/l, Lactate 1.1 mmol/l

Blood count: WBC 6.1 cells/mm³, Hb 12.6 g/dl, platelets 312000/mm³

Serum: creatinine 1.1mg/dl, GOT 44 U/l GPT 65 U/L, GGT 81 U/l, bilirubin 0.7 mg/dl, CRP 8.2 mg/dl, PCT 0.9 pg/ml

Patient directive: not mentioned

Relatives: maximum therapy

Plan: improve oxygenation, extubation planned

**CASE 4**

They care for 88-year-old retiree Emilia Konz (48kg, 159 cm) with femoral neck fracture.

Airway: dental prosthesis

Breathing: room air

Circulation: stable, hypertension on admission (180/100 mmHg)

Disability: mild dementia, sometimes confused

Exposure: none

Signs & Symptoms: tripping fall, thereby femur fracture

Allergies: none

Medication: Metroprolol, Gingko

Past Medical History: never seen a doctor

Last Meal: last night

Events: lives alone with 8 cats, negligence

CT thorax: not performed

Echocardiography: good LV function with significant LV hypertrophy and diastolic relaxation abnormality

ABG: pH 7.35 pO2 62 mmHg, pCO2 56 mmHg, SaO2 93%, Na 145 mmol/l K 3.8 mmol/l, lactate 0.9 mmol/l

Blood count: WBC 9,9 cells/mm³, Hb 10,4 g/dl, platelets 97000/mm³

Serum: creatinine 1.0mg/dl, GOT 122 U/l GPT 90 U/L, GGT 99 U/l, bilirubin 1.1 mg/dl, CRP 6.5 mg/dl, PCT 0.4 pg/ml

Patient directive: none

Relatives: none

Plan: femoral nailing at night, no recovery room currently available

**CASE 5**

You care for the 34-year-old car constructer Peter Jenz (90 kg, 188 cm) injured by a fall from a height of 10 meters with suicidal intent.

Airway: Intubated by emergency physician

Breathing: FiO2 80%, Pmax 30, Peep 10, pneumothorax on the right side

Circulation: Hemorrhagic shock due to splenic rupture and open femur fracture, high-dose norepinephrine therapy

Disability: RASS -5, anisocoria, signs of inhernation

Exposure: Intubated

Signs & Symptoms: onset of all symptoms after fall from roof of a house

Allergies: unknown

Medication: unknown, currently ketamine, sufentanil, rocuronium

Past Medical History: unknown

Last Meal: unknown

Events: none

CT chest: severe pulmonary contusions, rib series fractures bds.

Head CT: Diffuse hemorrhage in all areas of cerebrum and brainstem ganglia, incipient cerebral edema, epidural hematoma with midline shift and midbrain inherniation, suspected dens axis fracture

Trauma spiral: splenic rupture (IV), free fluid in abdomen, femur fracture bds, forearm fractures bds,vertebral fracture LWK 5 with complete transection of spinal cord

Echocardiography: moderate LV function with volume deficiency.

ABG: pH 7.2 pO2 99 mmHg, pCO2 32 mmHg, SaO2 99%, Na 149 mmol/l K 4.1 mmol/l, Lactate 11.1 mmol/l

Blood count: WBC 16,9 cells/mm³, Hb 6,2 g/dl, Thrombocytes 21920/mm³

Serum: Creatinine 0.7mg/dl, GOT 345 U/l GPT 74 U/L, GGT 50 U/l, Bilirubin 1.1 mg/dl, CRP 0.6 mg/dl, PCT 1.1 pg/ml

Patient directive: unknown

Relatives: unknown

Plan: emergency surgery (brain, abdomen)

**CASE 6**

You care for a 66-year-old female patient (69 kg, 158 cm) who collapsed on the street and was resuscitated for 45 minutes by lay people and the ambulance service.

Airway: Intubated by emergency physician

Breathing: FiO2 100%, Pmax 20, Peep 7

Circulation: myocardial pump failure in STEMI, high-dose norepinephrine therapy

Disability: wide pupils without reaction to light, no awakening after ROSC.

Exposure: hypothermia 31°C core body temperature

Signs & Symptoms: unknown, collapse on the road

Allergies: unknown

Medication: unknown, currently midazolam, sufentanil

Past Medical History: unknown

Last Meal: unknown

Events: none

CT chest: opaque infiltrates on both lungs

CT-head: not yet performed

Echocardiography: highly impaired LV function with akinesia of the anterior wall

ABG: pH 7.0 pO2 189 mmHg, pCO2 28 mmHg, SaO2 99%, Na 141 mmol/l K 5.6 mmol/l, lactate 16.9 mmol/l

Blood count: WBC 13.9 cells/mm³, Hb 11.2 g/dl, Platelets 170231/mm³

Serum: creatinine 0.8mg/dl, GOT 66 U/l GPT 56 U/L, GGT 51 U/l, bilirubin 0.8 mg/dl, CRP 1.6 mg/dl, PCT 1.5 pg/ml

Patient directive: unknown

Relatives: unknown

Plan: intervention planned by cardiology, cath-lab in 10 min.

**CASE 7**

You care for 67-year-old retiree Marius Althoff (110 kg, 187 cm, formerly a bricklayer) with COVID 19 infection.

[see case 1]

**CASE 8**

They care for 69-year-old retiree Manuel Geisser (90kg, 181 cm) with an intracranial hemorrhage.

Airway: Intubated for 12 days, tracheotomized 3 days ago.

Breathing: FiO2 45%, Pmax 22, Peep 6

Circulation: stable, rather hypertensive

Disability: only basal reaction, currently RASS -2, partly agitated, left hemiplegia

Exposure: central line, arterial line, tracheal tube

Signs & Symptoms: onset 12 days ago headache, hypertension, opacification, aspiration

Allergies: none

Medication: Enalapril (preexisting), ASA (preexisting), Clonidine (current), Piritramide (current), Piperacillin/Tazobactam (current), Pantoprazole (current), Enoxaparin low dose (current)

Past Medical History: condition after colon resection for carcinoma 8 years ago, Z.n prostate carcinoma 3 years ago.

Last Meal: enteral nutrition via gastric tube

Events: none

CT chest: pneumonic infiltrates, regressing

Echocardiography: good LV function, hypertrophy

ABG: balanced

Blood count: WBC 4.7 cells/mm³, Hb 10.4 g/dl, platelets 134000/mm³

Serum: Creatinine 1.3mg/dl, GOT 215 U/l GPT 177 U/L, GGT 220 U/l, Bilirubin 1.0 mg/dl, CRP 8.2 mg/dl, PCT 1.4 pg/ml

Patient directive: none

Relatives: maximum therapy, threaten physicians with legal issues

Plan: weaning, early rehabilitation transfer in 6 days

**CASE 9**

They are providing care for 28-year-old Luisa Hermanns (130 kg, 161 cm) with COVID 19 infection.

Airway: Intubated 14 days ago for ARDS.

Breathing: FiO2 100%, Pmax 30, Peep 15, intermittent prone positioning (difficult)

Circulation: shock situation

Disability: current RASS -5 due to sedation

Exposure: central venous line, arterial line, endotracheal tube

Signs & Symptoms: onset 18 days ago with high fever, infection via kindergarten child, 15 days ago significant respiratory distress, referral via ED for ARDS

Allergies: none

Medication: Norepinephrine (high-dose), Epinephrine (high dose), Vasopressin, Sufentanil, Midazolam, Rocuronium, Pantozol, Heparin low dose

Past medical history: severe obesity, asthma

Last Meal: parenteral nutrition for paralytic ileus with high gastric reflux

Events: none, multiple ulcerations on the back

CT chest: opaque infiltrates on both sides, basal atelectasis, bacterial pneumonic infiltrates on left side

Echocardiography: good LV function

ABG: pH 7.25 pO2 49 mmHg, pCO2 66 mmHg, SaO2 91%, Na 153 mmol/l K 5.8 mmol/l, Lactate 3.2 mmol/l

Blood count: WBC 16.2 cells/mm³, Hb 11.8 g/dl, Platelets 90156/mm³

Serum: creatinine 1.3mg/dl, GOT 264 U/l GPT 199 U/L, GGT 412 U/l, bilirubin 2.1 mg/dl, CRP 36 mg/dl, PCT 66.2 pg/ml

Patient directive: none

Relatives: maximum therapy

Plan: improve oxygenation, tracheostomy resolved, transfer to ECMO being evaluated, ECMO not yet available.

**CASE 10**

You care for a 44-year-old teacher Johannes Elker (68 kg, 182 cm) with COVID-19.

[See case 3]

**CASE 11**

You are caring for 51-year-old police officer Anja Homms (69 kg, 167 cm) with four-quadrant peritonitis after colon resection for carcinoma (pT3N2M0)

Airway: intubated

Breathing: ventilated, FiO2 65%, Pmax 23 mbar, PEEP 8 mbar.

Circulation: septic shock

Disability: deeply sedated

Exposure: mottled skin, fingers and toes with single necroses

Signs & Symptoms: 3 days after colon surgery sudden fever and leucocytosis, acute abdomen, surgical revision in OR, revision of anastomotic insufficiency, irrigation, lavage, 2x re-op so far, now vacuum therapy of abdomen

Allergies: Doxycycline

Medication: norepinephrine, propofol, sufentanil, pantozol,

Past Medical History: Caesarean section, Hypertension

Last Meal: enteral nutrition via gastric tube

Events: none

CT chest: no signs of pneumonia

Echocardiography: hypovolemia, signs of septic cardiomyopathy

ABG: balanced

Blood count: WBC 11.3 cells/mm³, Hb 11.1 g/dl, platelets 112000/mm³

Serum: Creatinine 6.1mg/dl, GOT 1651 U/l GPT 2154 U/L, GGT 1156 U/l, Bilirubin 4.9 mg/dl, CRP 22.5 mg/dl, PCT 18.1 pg/ml

Patient directive: none

Relatives: maximum therapy

Plan: start dialysis today (dialysis catheter already in place), stabilization, surgery tomorrow

**CASE 12**

They care for 68-year-old retiree Hajo Gromms (90kg, 181 cm) with with an intracranial haemorrhage

[see case 8]

**CASE 13**

You treat the 61-year-old industrial clerk Hermann Loss (100kg, 174 cm) with a 6.8 cm abdominal aortic aneurysm, which was discovered occasionally during a control examination. Due to several vascular surgical interventions on the femoral vessels, the indication for open BAA treatment was given.

The surgery is scheduled to take place tomorrow.

Airway: clear

Breathing: 92% on room air

Circulation: hypertensive

Disability: without restrictions

Exposure: no

Signs & Symptoms: Asymptomatic findings

Allergies: none

Medication: Metoprolol, Allopurinol, Metformin, Enalapril, ASA, Marcumar

Past Medical History: multiple bypass surgeries and revisions, COPD GOLD I, Obstructive Sleep Apnea Syndrome, NIDDM

Last Meal: 1 hour ago

Events: none

CT chest: none

Echocardiography: good LV function, hypertrophy.

ABG: chronic, metabolically compensated respiratory acidosis

Blood count: WBC 6.7 cells/mm³, Hb 12.4 g/dl, platelets 256000/mm³

Serum: creatinine 1.4mg/dl, GOT 200 U/l GPT 124 U/L, GGT 201 U/l, bilirubin 1.1 mg/dl, CRP 2.2 mg/dl, PCT 1.0 pg/ml

Patient directive: none

Relatives: maximum therapy

Plan: surgery tomorrow with peridural anesthesia, one to two days intensive monitoring

**CASE 14**

You care for 31-year-old Emilia Sterrer, who suffered a severe polytrauma 5 years ago. Since the accident, she has been severely disabled. Now she is admitted due to a dysfunction of the ventriculo-peritoneal shunt and is to be operated. Due to epileptic seizures and the provision of a tracheostoma, transfer to the intensive care unit for monitoring is necessary.

Airway: tracheostoma, cannula unblocked

Breathing: 96% on room air

Circulation: without path. Findings

Diasbility: apallic syndrome, baclofen pump for severe tetra spasticity, epileptic seizures (2x/d)

Exposure: Port, percutaneous gastric tube, tracheostoma, baclofen pump

Signs & Symptoms: Increasing intracranial pressure signs in the last days

Allergies: Penicillin, aspirine

Medication: levetiracetam, valproate, bromine derivative, pantoprazol,

Past Medical History: severe SHT with diffuse axonal shear trauma several years ago

Last Meal: 2 hours ago

Events: none

CT chest: none

Echocardiography: none

ABG: balanced

Blood count: WBC 4.2 cells/mm³, Hb 13.4 g/dl, platelets 199000/mm³

Serum: Creatinine 0.3mg/dl, GOT 16 U/l GPT 35 U/L, GGT 33 U/l, Bilirubin 0.4 mg/dl, CRP 0.2 mg/dl, PCT 0.2 pg/ml

Patient directive: none

Relatives: maximum therapy

Plan: surgery tomorrow

**CASE 15**

You care for Harald Jescher, a 61-year-old pharmacist with amyotrophic lateral sclerosis (ALS). He suffers acute respiratory failure in the context of bacterial pneumonia with pre-existing severe respiratory insufficiency.

Airway: free, CPAP device

Breathing: 91% on room air, AF 34/min, significant respiratory distress, secretion retention.

Circulation: without path. Findings

Diasbility: flaccid tetraplegia, dysphagia

Exposure: PEG

Signs & Symptoms: Increasing dyspnoea, purulent sputum, which can hardly be coughed up

Allergies: none

Medication: none

Past Medical History: ALS for 3 years

Last Meal: 2 hours ago via PEG

Events: none

CT-Thorax: pneumonic infiltrates bds., no evidence for COVID

Echocardiography: ok

ABG: pH 7.2, CO2 56 mmHg, O2 45 mmHg, SaO2 91%, HCO3 15 mmoL/l, lactate 4.3 mmol/dl

Blood count: WBC 17,2 cells/mm³, Hb 12,1 g/dl, platelets 456000/mm³

Serum: creatinine 0.3mg/dl, GOT 19 U/l GPT 28 U/L, GGT 66 U/l, bilirubin 0.6 mg/dl, CRP 25.2 mg/dl, PCT 6.3 ng/ml.

Patient directive: none

Relative: maximal therapy (still wants to write his books with eye control).

Plan: decision making, sepsis therapy, if necessary, tracheostomy

**CASE 16**

They care for Jens Klein, a 38-year-old patient with Down syndrome who lives in a long-term care facility. He is admitted to the emergency department with COVID 19

Airway: not secured, partially obstructed, Wendl tube (naso-pharyngeal tube)

Breathing: 92% on room air, AF 36/min.

Circulation: Hypertensive

Disability: Delirious, anxious-aggressive/exhausted, previously probably always friendly with good quality of life, works in sheltered workshop

Exposure: none

Signs & Symptoms: Increasing shortness of breath since 3 days, infected by COVID-positive nurse

Allergies: none

Medication: Metoprolol

Past Medical History: Atrial and ventricular septal defect, occlusion in infancy, hypertension

Last Meal: unknown

Events: none

CT chest: opaque glass infiltrates

Echocardiography: left ventricular hypertrophy

ABG: pH 7.1, CO2 99 mmHg, O2 42 mmHg, SaO2 86%, HCO3 20 mmoL/l, lactate 6.3 mmol/dl

Blood count: WBC 17,2 cells/mm³, Hb 14,1 g/dl, Thrombocytes 391050/mm³

Serum: creatinine 0.9mg/dl, GOT 44 U/l GPT 66 U/L, GGT 61 U/l, bilirubin 0.9 mg/dl, CRP 18.1 mg/dl, PCT 0.9 ng/ml.

Patient directive: none

Relatives: caregiver (currently unavailable).

Plan: decision making

**Questions on all cases**

**What score do you give the patient related to expected survival under currently possible intensive care therapy?**

Very likely (0 points)

Probable (5 points)

Intermediate (15 points)

Unlikely (30 points)

**What score do you give the patient related to the best rehabilitation potential regarding autonomous volition?**

Complete autonomy (0 points)

Predominant autonomy (5 points)

Predominant heteronomy (15 points)

Complete heteronomy (30 points)

**What score do you give the patient in terms of days left in the ICU?**

1-2 days (0 points)

3-9 days (5 points)

10-25 days (15 points)

more than 25 days (30 points)

**Open Question at the end of the survey**

Comments on the score

1. is the use of the probability of survival in this score morally/factually right for you?

2. Is the use of the prospect of self-determination in this score morally/factually right for you?

3. is the use of the days of intensive care still expected in this score morally/factually right for you?
